# Exercise-induced ventricular tachycardia in a young adult: what lies beneath?

**DOI:** 10.1007/s12471-026-02037-7

**Published:** 2026-04-10

**Authors:** Ana Sofia Nogueira Fernandes, Rui Pedro Files Flores, Sérgia Andreia Alves Rodrigues da Rocha Costa

**Affiliations:** Department of Cardiology, ULS Braga, Braga, Portugal

A previously healthy 24-year-old man presented to the emergency department after experiencing palpitations, dizziness, and dyspnoea during a football match. Upon admission, he was hemodynamically stable without clinical signs of heart failure. The initial 12-lead electrocardiogram showed a wide-complex tachycardia at 224 bpm, with left bundle branch block morphology, superior axis, and late R‑wave transition in the precordial leads (Fig. 1). Electrical cardioversion successfully restored sinus rhythm, which revealed T‑wave inversion in inferior and lateral precordial leads (Fig. 2).

**Fig. 1:**
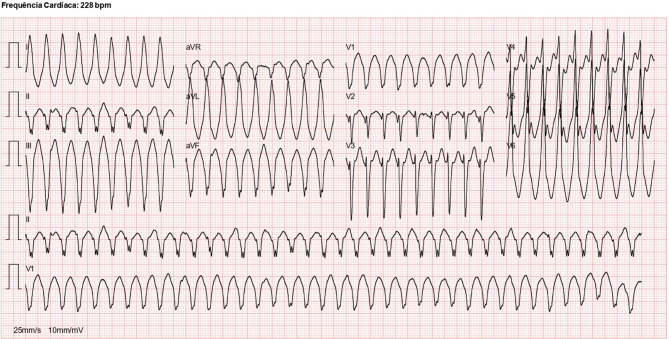
Twelve-lead ECG showing wide complex tachycardia at 224 bpm, with left bundle branch block morphology and inferior axis

**Fig. 2:**
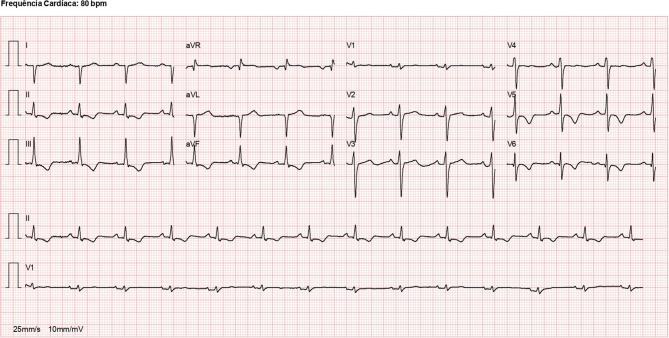
Twelve-lead ECG in sinus rhythm showing T‑wave inversion in inferior and lateral precordial leads (V5–V6)

*What is the most likely underlying diagnosis in this young patient with exercise-induced sustained monomorphic ventricular tachycardia, and which investigations are essential to confirm it?*

## Answer

You will find the answer elsewhere in this issue.

